# Nichtinanspruchnahme medizinischer Leistungen in der COVID-19-Pandemie bei Personen mit chronischen Erkrankungen

**DOI:** 10.1007/s00103-023-03665-9

**Published:** 2023-02-01

**Authors:** Ines Schäfer, Alena Haack, Marie Neumann, Uwe Koch-Gromus, Martin Scherer, Elina Petersen

**Affiliations:** 1 Epidemiologisches Studienzentrum, Universitätsklinikum Hamburg-Eppendorf, 2. OG, Christoph-Probst-Weg 3, 20251 Hamburg, Deutschland; 2 Universitäres Herz- und Gefäßzentrum, Universitätsklinikum Hamburg-Eppendorf, Hamburg, Deutschland; 3 Institut und Poliklinik für Allgemeinmedizin, Universitätsklinikum Hamburg-Eppendorf, Hamburg, Deutschland

**Keywords:** COVID-19, Pandemiefolgen, Inanspruchnahme, Chronische Erkrankungen, Vulnerable Gruppen, COVID-19 pandemic, Pandemic consequences, Healthcare utilization, Chronic disease, Vulnerable groups

## Abstract

**Einleitung:**

Die COVID-19-Pandemie wirkt sich auch auf die medizinische Versorgung anderer Erkrankungen aus. Differenziert zwischen patient:innen- und anbieter:innenseitigen Gründen wurde untersucht, inwieweit Personen mit chronischen Erkrankungen vom Aussetzen medizinischer Versorgungsleistungen betroffen sind.

**Methoden:**

Es wurde eine Querschnittstudie auf Datenbasis der Kohortenstudie Hamburg City Health Study (HCHS) durchgeführt. Die Studienpopulation bestand aus allen HCHS-Teilnehmer:innen (Stichprobe der Bevölkerung Hamburgs, 45–74 Jahre) zwischen April 2020 und November 2021. Über das „COVID-19-Modul“ der HCHS wurde die Inanspruchnahme von Versorgungsleistungen erhoben. Als Grunderkrankungen wurden u. a. Herz-Kreislauf‑, Nieren- und Lungenerkrankungen, Krebs und Diabetes mellitus betrachtet. Die Daten wurden deskriptiv und multivariat in logistischen Regressionen ausgewertet.

**Ergebnisse:**

Von 2047 Teilnehmer:innen hatten 47,9 % mindestens eine Vorerkrankung. 21,4 % der Personen mit Vorerkrankungen hatten mindestens eine Versorgungsleistung ausgesetzt oder eine Terminabsage erhalten. 15,4 % gaben an, von sich aus auf einen Arztbesuch verzichtet zu haben. Fachärztliche Leistungen (Anteil 43,8 %) entfielen häufiger als hausärztliche (16,6 %). Nach Adjustierung für Alter, Geschlecht und Bildung erwiesen sich Lungen- (OR 1,80; *p* < 0,008) und Krebserkrankungen (OR 2,33; *p* < 0,001) als unabhängige Risikofaktoren für ärztliche Terminabsagen. 42,2 % der patient:innenseitigen Absagen erfolgten aus Angst vor einer Ansteckung mit SARS-CoV‑2.

**Diskussion:**

Gesundheitspolitik und Medien stehen vor der Herausforderung, mit Ängsten in der Bevölkerung vor einer Infektion so umzugehen, dass notwendige Versorgungsleistungen dadurch nicht vermieden werden.

**Zusatzmaterial online:**

Zusätzliche Informationen sind in der Online-Version dieses Artikels (10.1007/s00103-023-03665-9) enthalten.

## Abstract (en)

**Introduction:**

The COVID-19 pandemic is also impacting the medical care for other diseases. The extent to which people with chronic diseases are affected by the suspension of medical services is investigated—differentiating between patient and provider perspectives.

**Method:**

A cross-sectional study was conducted based on data from the longitudinal Hamburg City Health Study (HCHS). The study population was all HCHS participants (a sample of the population of Hamburg, 45–74 years) between April 2020 and November 2021. Utilisation of medical services was collected via the “COVID-19-module” of the HCHS. The chronic conditions included cardiovascular disease, kidney and lung disease, cancer and diabetes mellitus; analyses were descriptive and multivariate.

**Results:**

Of the 2047 participants, 47.9% had at least one previous illness. Of those with pre-existing conditions, 21.4% had at least one healthcare service suspended or an appointment cancelled. In addition, 15.4% stated that they decided by themselves not to attend a doctor’s appointment. Specialist care services (43.8% of all cancellations) were cancelled more frequently than general practitioner care (16.6%). After adjustment for age, gender and education, lung disease (OR 1.80; *p* < 0.008) and cancer (OR 2.33; *p* < 0.001) were found to be independent risk factors for appointment cancellations by healthcare providers. Of cancellations by patients, 42.2% were due to their fear of an infection with the coronavirus SARS-CoV‑2.

**Discussion:**

Health policy and the media are faced with the challenge of dealing with fears of infection in the population in such a way that they do not lead to an avoidance of necessary care services.

## Einleitung

Die COVID-19-Pandemie hat global zu einer hohen Belastung der Gesundheitssysteme geführt. Neben der zentralen Aufgabe der Versorgung von COVID-19-Erkrankten haben bevölkerungsbezogene Maßnahmen zur Eindämmung des Infektionsgeschehens potenzielle Auswirkungen auch auf die gesundheitliche Versorgung anderer Erkrankungen. Allgemeine Kontaktbeschränkungen sowie die Befürchtung, sich mit dem Coronavirus SARS-CoV‑2 zu infizieren, beeinflussen aufseiten der Patient:innen die Entscheidung, eine medizinische Versorgungseinrichtung aufzusuchen. Seitens der Leistungsanbieter:innen kann sich neben diesen Faktoren auch die vom Bund und den Ländern ausgegebene Aufforderung, planbare bzw. elektive Maßnahmen in bestimmten Pandemiephasen hinauszuschieben, auf das Leistungsangebot auswirken und evtl. Terminabsagen zur Folge haben. Für die ambulante Versorgung bedeutete u. a. die Einrichtung von Infektsprechstunden eine zusätzliche Umstrukturierung und ggf. Kapazitätseinschränkung des Behandlungsangebotes [1].

In Deutschland ist die Zahl der Behandlungsfälle in der ambulanten sowie in der stationären Versorgung insbesondere zu Beginn der COVID-19-Pandemie gesunken [2, 3]. Als Ursachen werden sowohl eine Einschränkung der Regelversorgung als auch eine geringere Inanspruchnahme angeführt [4, 5]. Diese Differenzierung in „Angebot und Nachfrage“, die auf unterschiedliche Determinanten verweist, wurde bisher in Studien zu Personen mit Vorerkrankungen nicht explizit berücksichtigt.

Vulnerable Bevölkerungsgruppen sind besonders stark von Krisen betroffen, die das Gesundheitssystem beeinflussen [6]. Zu diesen Gruppen zählen neben Personen mit niedrigem sozioökonomischen Status, älteren Menschen und Migrant:innen vor allem Personen mit schweren oder chronischen Erkrankungen, die in der vorliegenden Studie im Fokus stehen. In Deutschland leiden ca. 40 % der Bevölkerung an mindestens einer chronischen Erkrankung, in höheren Altersgruppen über 60 % [7]. Die Pandemie bzw. umfassende Eindämmungsmaßnahmen können sich für vorerkrankte Personen nicht nur in Hinblick auf einen schwereren Verlauf einer SARS-CoV-2-Infektion, sondern ganz unmittelbar auf die Kontinuität und die Qualität der Versorgung ihrer Grunderkrankung auswirken. Mögliche Folgen des Aussetzens medizinischer Versorgungsleistungen auf Ebene der Sekundärprävention, der Behandlung sowie des Disease-Managements sind eine beschleunigte Progression und eine erhöhte Komplikationsrate der Grunderkrankung. Die Deutsche Gesellschaft für Allgemeinmedizin sowie weitere medizinische Fachgesellschaften betonten früh die Notwendigkeit, bei der Anpassung des Gesundheitssystems an die Erfordernisse der COVID-19-Pandemie die Sicherstellung der gesundheitlichen Versorgung von vulnerablen Bevölkerungsgruppen nicht aus den Augen zu verlieren [8].

Hinweise darauf, dass in der COVID-19-Pandemie ein Rückgang der Versorgungsleistungen auch bei Personen mit chronischen Erkrankungen zu verzeichnen ist, ergeben sich aus der internationalen Literatur [9] und für Deutschland u. a. aus der rückläufigen Anzahl spezifischer stationärer und ambulanter Behandlungsfälle [2, 10, 11].

Während die Belastung und die Herausforderungen, die COVID-19 für das Gesundheitssystem insgesamt darstellt, präsent und gut dokumentiert sind, ist über die damit verbundenen Auswirkungen auf die Versorgung chronischer Krankheiten weniger bekannt [12]. Bisher sind wenige Quellen verfügbar, die unter Pandemiebedingungen auf Bevölkerungsebene einen direkten Vergleich der Inanspruchnahme bzw. des Versorgungsangebots bei Personen mit und ohne chronische Erkrankungen erlauben.

### Studienziel

Die Studie fokussiert auf die Nichtinanspruchnahme von Versorgungsleistungen sowie auf deren Gründe und Initiator:innen in der COVID-19-Pandemie. Im Einzelnen sollte untersucht werden:

1. In welchem Ausmaß waren Personen mit vorbestehenden Erkrankungen in der COVID-19-Pandemie vom Aussetzen medizinischer Leistungen betroffen?
2. Besteht ein Unterschied zwischen Personen mit und ohne Vorerkrankungen hinsichtlich der Nichtinanspruchnahme bzw. des Aussetzens medizinischer Leistungen?
3. Inwieweit war die Absage bzw. Verschiebung der medizinischen Leistung initiiert durch die Patient:innen selbst (Nichtinanspruchnahme) und inwieweit durch die Anbieter:innen von Versorgungsleistungen?
4. Was waren die Gründe für die Nichtinanspruchnahme bzw. Terminabsagen?

## Methoden

Datenbasis für die Analysen ist die Hamburg City Health Study (HCHS) eine longitudinale, populationsbezogene Beobachtungsstudie, die seit 2016 am Universitätsklinikum Hamburg-Eppendorf (UKE) durchgeführt wird. In einer Kooperation von über 30 UKE-Kliniken und Instituten werden multidisziplinär zahlreiche Risikofaktoren für „Volkskrankheiten“, wie u. a. Herzinfarkt, Schlaganfall, Krebs, Demenz und Depression, erhoben und in ihrem Zusammenwirken analysiert. Basierend auf einer nach Alter und Geschlecht stratifizierten Zufallsstichprobe des Einwohnermeldeamtes werden in die HCHS Personen aus der Allgemeinbevölkerung eingeschlossen, die zum Zeitpunkt des Studieneinschlusses Einwohner:in der Stadt Hamburg und 45 bis 74 Jahre alt sind. Ausschlusskriterien sind nicht ausreichende (sprachliche oder kognitive) Fähigkeiten zum Ausfüllen der Fragebögen sowie (körperliche) Einschränkungen, die das Absolvieren des siebenstündigen Untersuchungsprogramms im Studienzentrum nicht erlauben. Derzeit (Stand August 2022) sind mehr als 17.000 Teilnehmer:innen (TN) untersucht worden.

Mit Beginn der Pandemie wurde vor dem Hintergrund des gesundheitspolitischen und wissenschaftlichen Potenzials der HCHS zwischen der Freien und Hansestadt Hamburg und einem interdisziplinären Konsortium des UKE im April 2020 eine Kooperation vereinbart, die eine Abschätzung der SARS-CoV-2-Seropositivität in der Bevölkerung auf Basis der HCHS leistete. Im Rahmen dieses Förderprojektes wurden spezifische Fragestellungen (COVID-19-Modul der HCHS) sowie SARS-CoV-2-Antikörpertests für alle konsekutiven TN in die HCHS integriert.

### Methodik der Analysen zur Inanspruchnahme

#### Studiendesign und Population.

Bei der vorliegenden Arbeit handelt es sich um eine querschnittliche Beobachtungsstudie. Alle konsekutiven HCHS-Teilnehmer:innen im Zeitraum 01.04.2020 bis 21.11.2021 erhielten den COVID-19-Fragebogen; die Ein- und Ausschlusskriterien entsprachen denen der HCHS (s. Datenbasis).

#### Datenerhebung.

Im Rahmen des HCHS-Untersuchungsprogramms und über umfangreiche Fragebogen werden detaillierte Informationen zu Erkrankungen sowie zu klinischen und psychosozialen Parametern generiert; die Methodik und Inhalte der HCHS sind an anderer Stelle detailliert beschrieben [13].

Der COVID-19-Fragebogen umfasst u. a. anamnestische Fragen zu PCR-Tests, COVID-19-Symptomen, psychosozialen Items sowie zum Inanspruchnahmeverhalten und wird online oder auf Papier ausgefüllt.

Die *Fragen zur Inanspruchnahme gesundheitlicher Leistungen* während der COVID-19-Pandemie entsprechen zum Teil denen des vom Robert Koch-Institut (RKI) durchgeführten Projektes „Corona-Monitoring lokal“ (CoMoLo; [14]). Erfragt wurde, ob die TN selbst auf einen Arztbesuch verzichtet hatten, obwohl dieser geplant war oder sie Beschwerden hatten. Falls dies bejaht wurde, wurde nachfolgend erfasst, welche Termine nach Fachrichtungen (Hausarzt, Facharzt, Zahnarzt) ausgesetzt wurden und ob trotz eines medizinischen Notfalls keine Rettungsstelle/Notaufnahme aufgesucht wurde.

In einem zweiten Block wurde erfragt, ob ein geplanter Arztbesuch seitens der Leistungserbringer:innen abgesagt oder verschoben wurde. Falls ja, sollte angegeben werden, ob es sich dabei um fachärztliche, hausärztliche oder zahnärztliche Termine bzw. um Krankenhausbehandlungen/Operationen, stationäre Rehamaßnahmen, psychotherapeutische oder Heilbehandlungen handelte. Für beide Blöcke wurden die Gründe der Nichtinanspruchnahme als Freitextangabe erhoben. Als Beginn des Referenzzeitraums für die Angaben zur Inanspruchnahme wurde Februar 2020 vorgegeben.

In die hier betrachteten Gruppen der überwiegend chronischen* Erkrankungen* wurden Herz-Kreislauf-Erkrankungen, Nieren- und Lungenerkrankungen, Krebs, Diabetes mellitus, Schlaganfall und Demenz eingeschlossen. Diese Diagnosen gehen vielfach mit dem Bedarf an einer kontinuierlichen medizinischen Versorgung bzw. einem regelmäßigen Monitoring einher. Darüber hinaus werden für die Betroffenen häufiger schwere Verläufe einer COVID-19-Erkrankung beobachtet [15, 16]. In der HCHS wurden die Diagnosen über anamnestische Fragen und/oder klinische Parameter definiert (Onlinematerial 1). Es handelt sich dabei um standardisierte und durch die jeweiligen Fachdisziplinen evaluierte Diagnosen der HCHS. Somit begründet sich die Auswahl der hier betrachteten Diagnosen auch in der Verfügbarkeit valider Daten. Aus den Angaben zum Vorliegen der einzelnen Erkrankungen wurde die dichotome (Ja/Nein) Variable „mindestens eine vorbestehende ärztlich diagnostizierte Erkrankung“ generiert.

Schulische und berufliche Bildungsabschlüsse wurden im Basisfragebogen der HCHS erfasst und anhand der International Standard Classification of Education (ISCED; [17]) kategoriell in 3 Bildungsgruppen unterteilt.

### Statistische Analysen

Nach der Datenbereinigung wurden quantitative Datenauswertungen mittels der Statistiksoftware R (Version 4.2.1) durchgeführt. Die Gruppenaufteilung erfolgte nach (i) mindestens einer Vorerkrankung und (ii) keiner Vorerkrankung. Für den deskriptiven Vergleich wurden für kategoriale Variablen absolute und relative Häufigkeiten angegeben und mittels Chi-Quadrat-Tests verglichen. Stetige Variablen wurden als Mittelwerte und Standardabweichungen bzw. Median und Interquartilsabstände angegeben. Das Signifikanzniveau lag bei α < 0,05. Im logistischen Regressionsmodell wurde das Aussetzen medizinischer Leistungen in Abhängigkeit von vorbestehenden Erkrankungen adjustiert für Alter, Geschlecht und Bildungsstatus analysiert und Odds Ratios, 95 %-Konfidenzintervalle sowie *p*-Werte berichtet und mittels Forest-Plots visualisiert. Die Regressionen wurden dabei als Complete-Case-Analyse durchgeführt.

## Ergebnisse

Der COVID-19-Fragebogen wurde an alle 2566 TN ausgegeben, die zwischen April 2020 und November 2021 in die HCHS eingeschlossenen wurden. Die vorliegenden Auswertungen basieren auf den Daten der 2047 TN (79,7 %), die im COVID-19-Fragebogen Angaben zur Nichtinanspruchnahme von Versorgungsleistungen gemacht haben (Tab. 1). Das Durchschnittsalter lag bei 55,3 Jahren (SD 6,8) und der Anteil weiblicher TN bei 45,2 %. Die TN wurden im Median 13,7 Monate nach dem Bezugszeitpunkt im Februar 2020 in die HCHS eingeschlossen (Interquartilsabstand 6,5; 17,8 Monate).

**Table 1:** 

	Gesamt *n* (%)	Personen mit mind. einer Vorerkrankung *n* (%)	Personen ohne Vorerkrankung *n* (%)
*Gesamt*	2047 (100,0)	857 (41,9)	1190 (58,1)
*Geschlecht*			
Frauen	926 (45,2)	406 (47,4)	520 (43,7)
Männer	1121 (54,8)	451 (52,6)	670 (56,3)
*Alter *(Jahre)			
Mittelwert (SD)	55,33 (6,8)	56,82 (7,1)	54,26 (6,3)
Median	54 (50; 6)	55 [51; 6]	53 [49; 58]
*Bildung*			
Hoch	1062 (52,5)	408 (48,3)	654 (55,5)
Mittel	894 (44,2)	397 (47,0)	497 (42,2)
Niedrig	66 (3,2)	39 (4,6)	27 (2,3)
*Nichtinanspruchnahme durch Patient:in trotz Beschwerden/Termin* (gültige *n* = 2046)	313 (15,3)	132 (15,4)	181 (15,2)
*Terminabsage durch Leistungserbringer:in* (gültig *n* = 2035)	150 (7,4)	85 (10,0)*	65 (5,5)
*Nichtinanspruchnahme oder Terminabsage durch Patient:in oder Leistungserbringer:in* (gültig *n* = 2047)	405 (19,8)	183 (21,3)	222 (18,7)

*p < 0,05

Bei 857 TN (41,9 %) bestand mindestens eine Vorerkrankung. In dieser Gruppe lag das mittlere Alter mit 57 Jahren etwas höher als in der Studienpopulation insgesamt. Männer und Personen mit mittlerem oder niedrigem Bildungsabschluss hatten häufiger Vorerkrankungen als Frauen und Personen der höchsten Bildungsgruppe. Den größten Anteil (38,4 %) an den hier erfassten Diagnosen machte die relativ breit gefasste Gruppe der Lungenerkrankungen mit einer Prävalenz von 16,2 % aus. Herz- und Nierenerkrankungen sowie Krebserkrankungen entsprachen jeweils ca. einem Viertel aller eingeschlossenen Diagnosen, für diese Erkrankungen lag die Prävalenz bei jeweils 10 % (Onlinematerial 1). Lungen‑, Nieren und Krebserkrankungen wurden häufiger von Frauen, Herzkrankheiten und Diabetes häufiger von Männern berichtet.

In der Studienpopulation haben seit Februar 2020 ca. 20 % aller TN mindestens eine gesundheitliche Versorgungsleistung ausgesetzt bzw. eine Terminabsage erhalten (Tab. 1). Dabei gaben insgesamt 15,3 % aller Befragten an, während der COVID-19-Pandemie mindestens einmal von sich aus auf einen Arztbesuch verzichtet zu haben, obwohl dieser geplant war oder sie Beschwerden hatten. Dieser Anteil war nahezu identisch in den Gruppen mit und ohne Vorerkrankungen. Eine Absage/Verschiebung seitens der Leistungserbringer:innen berichteten mit 10,0 % signifikant mehr Personen mit als ohne Vorerkrankungen (5,5 %; *p* < 0,001).

Im Regressionsmodell wurde geprüft, inwieweit das Vorliegen einer chronischen Erkrankung eigenständig mit dem Aussetzen gesundheitlicher Versorgungsleistungen assoziiert ist. Dazu wurden die mit einer Komorbidität und dem Inanspruchnahmeverhalten assoziierten potenziellen Confounder Alter, Geschlecht und Bildungsstatus in das Modell aufgenommen [18]. Das Modell der patient:innenseitigen Nichtinanspruchnahme umfasste in der Complete-Case-Analyse *n* = 1736 TN und das der Absagen durch Versorgungseinrichtungen *n* = 1730 TN.

**Figure 1:**
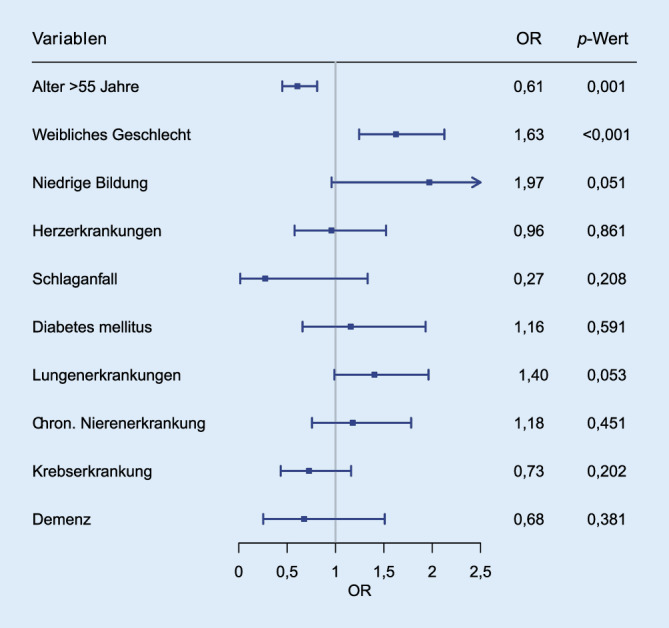

Die logistische Regression zeigte, dass Frauen und Personen der jüngeren Altersgruppen in der COVID-19-Pandemie signifikant seltener eine Versorgungseinrichtung aufsuchten als Männer bzw. Ältere (Abb. 1). Ein niedriger Bildungsstatus verdoppelte die Wahrscheinlichkeit der Nichtinanspruchnahme, allerdings war dieser Zusammenhang nicht signifikant.

**Figure 2:**
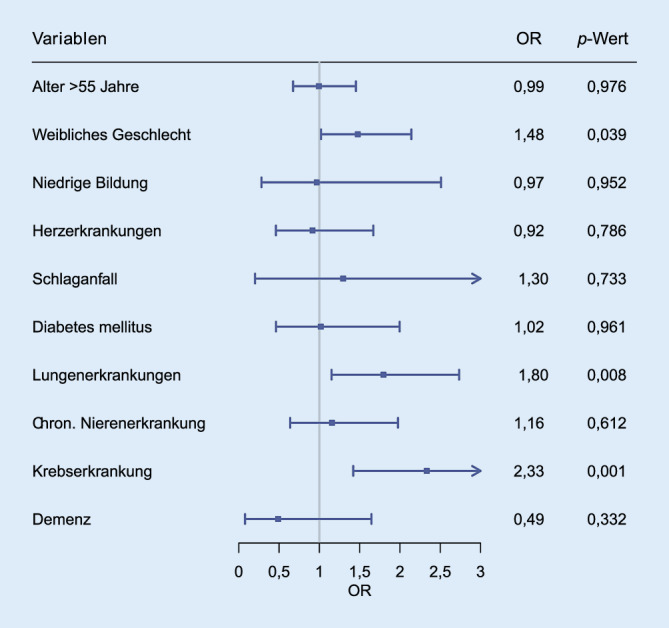

Im Unterschied zur patient:inneninitiierten Nichtinanspruchnahme zeigte sich, dass Terminabsagen durch die Versorgungseinrichtungen bei Personen mit chronischen Lungenerkrankungen (OR 1,80; *p* = 0,008) und Krebserkrankungen (OR 2,33; *p* < 0,001) signifikant häufiger waren (Abb. 2). Ebenso erfuhren Frauen signifikant häufiger eine Terminabsage als Männer (OR 1,48; *p* = 0,039). Das Alter war hier – anders als bei den patient:innenseitig ausgesetzten Terminen – nicht mit einer Terminabsage assoziiert.

### Subgruppenanalyse für Personen mit Vorerkrankungen: Fachrichtungen und Gründe der ausgesetzten Versorgungsleistungen

**Figure 3:**
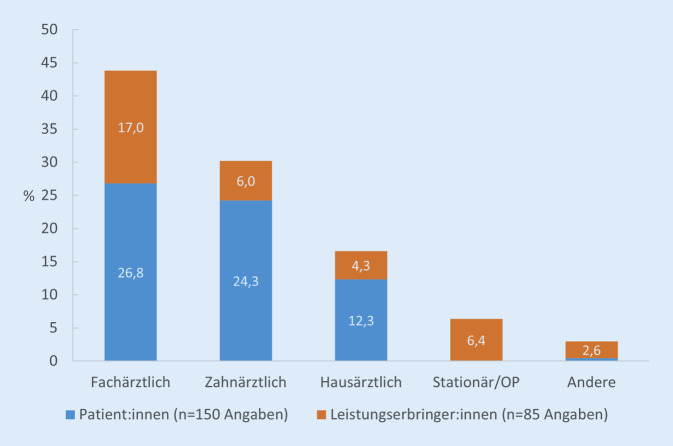

Ambulante Versorgungsleistungen bei Fach‑, Zahn- oder Hausärzt:innen wurden jeweils häufiger von den Patient:innen selbst als von den Praxen abgesagt (Abb. 3). Insgesamt lagen 235 Angaben zu den Fachrichtungen bei ausgesetzten Leistungen vor, von denen mit 43,8 % aller Angaben fachärztliche Leistungen sowohl patient:innen- als auch ärzt:innenseitig am häufigsten genannt wurden. 24,3 % aller abgesagten Termine waren auf patient:innenseitige Absagen von Zahnarztterminen zurückzuführen. Ausgesetzte Termine in der hausärztlichen Praxis machten 16,6 % aller Ausfälle aus und waren nur zu einem geringen Teil (4,3 %) initiiert durch die Praxis selbst. 6,4 % waren auf die Absage bzw. Verschiebungen einer stationären Behandlung seitens des Krankenhauses zurückzuführen. Notfallbehandlungen, stationäre Rehabilitationsmaßnahmen sowie psychotherapeutische oder Heilbehandlungen trugen mit insgesamt 2,6 % zu den ausgefallenen Versorgungsleistungen bei.

**Figure 4:**
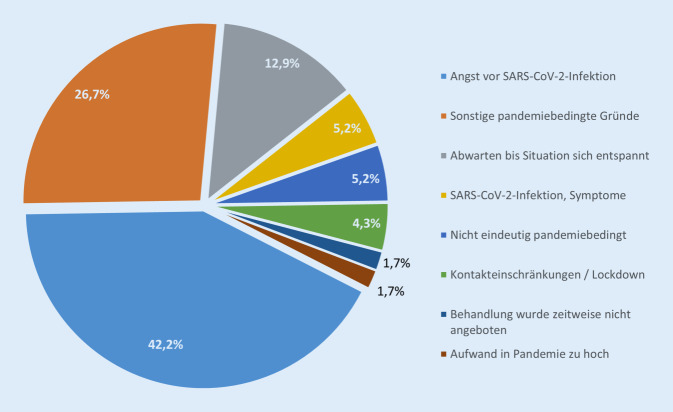

Befragt nach den Gründen für die Nichtinanspruchnahme (offene Frage) wurde von den TN am häufigsten die Angst vor einer Ansteckung mit dem Coronavirus SARS-CoV‑2 genannt (Abb. 4; 42,2 % aller patient:innenseitigen Absagen). 26,7 % gaben nicht näher spezifizierte pandemiebedingte Gründe an. Knapp 13 % wollten den Termin verschieben und zunächst abwarten, bis sich die Pandemielage „entspannt“.

**Figure 5:**
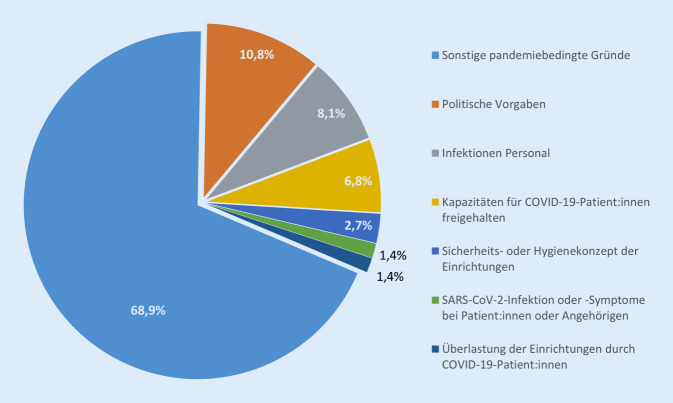

Die Gründe, die den Patient:innen seitens der Versorgungseinrichtungen genannt wurden, bezogen sich überwiegend allgemein auf die COVID-19-Pandemie (68,9 %; Abb. 5). Hinweise darauf, dass politische Vorgaben zur Zurückstellung aufschiebbarer Behandlungen der Absagegrund gewesen sein könnten, wurden in 10,8 % der Fälle genannt. In knapp 7,0 % wurde der Termin aufgrund von Erkrankungen des Personals abgesagt.

## Diskussion

In der Gruppe der Personen mit chronischen Erkrankungen war jede:r Fünfte (21,4 %) vom Aussetzen medizinischer Leistungen in der COVID-19-Pandemie betroffen.

### Vergleich der Ergebnisse mit denen anderer Studien

Personen mit einer chronischen Grunderkrankung standen bisher selten im Fokus der Analysen zu den Auswirkungen der COVID-19-Pandemie auf die Gesundheitsversorgung der Bevölkerung und die wenigen dazu vorliegenden Daten beziehen sich größtenteils auf die stationäre Versorgung einzelner Erkrankungen oder berücksichtigen Komorbidität als Covariate [19]. Scheidt-Nave et al. [2] gingen in einer frühen Übersichtsarbeit aus dem Jahr 2020 der Frage nach der Versorgungssituation von chronisch kranken Menschen in Deutschland nach und berichten sowohl für den stationären als auch für den ambulanten Bereich im Frühjahr 2020 einen deutlichen Rückgang unter anderem von onkologischen und kardiologischen Behandlungsfällen sowie von Behandlungsfällen zu Diabetes mellitus und psychischen Störungen. Weitere nationale Studien weisen jedoch darauf hin, dass auch in der COVID-19-Pandemie Personen mit chronischen Erkrankungen die notwendigen Versorgungsleistungen in Anspruch genommen haben [20]. Beispielsweise ging für Menschen mit Diabetes im Frühjahr 2020 die fachärztliche, jedoch nicht die allgemeinärztliche Inanspruchnahme vorübergehend zurück [21]. Ein globaler Survey der Weltgesundheitsorganisation (WHO; [22]) zeigte für drei Viertel der Länder weltweit relevante Einschränkungen der Regelversorgung von nichtübertragbaren Krankheiten als Folge der COVID-19-Pandemie.

Im multizentrischen CoMoLo-Projekt des RKI [14] gab ca. ein Drittel der Befragten zwischen 18 und 99 Jahren an, während der COVID-19-Pandemie auf einen Arztbesuch verzichtet zu haben, obwohl Beschwerden vorhanden waren oder der Besuch terminiert war. In der vorliegenden Studie lag dieser Anteil bei Personen mit und ohne Vorerkrankungen mit jeweils ca. 15 % deutlich niedriger.

### Unterschiede zwischen Personen mit und ohne Vorerkrankungen

In der vorliegenden Arbeit war die patient:inneninitiierte Nichtinanspruchnahme nicht signifikant mit einzelnen chronischen Grunderkrankungen assoziiert. Dies entspricht den Ergebnissen einer südkoreanischen Studie [6] sowie der Studie von Splinter et al. [18], in denen die Inanspruchnahmerate zwar vom wahrgenommenen Gesundheitszustand, aber nicht maßgeblich von vorbestehenden Erkrankungen beeinflusst wurde. In einem bundesweiten Online-Survey [19] wurde allerdings ein Rückgang der Versorgungsleistungen von Gesundheits-Check-ups um ca. 16,0 % ermittelt, wobei eine vorbestehende chronische Erkrankung einen signifikanten Risikofaktor darstellte.

Eine Terminabsage durch die Leistungsanbieter:innen hingegen war für Patient:innen mit chronischen Lungenerkrankungen und Krebserkrankungen doppelt so wahrscheinlich wie für Personen ohne diese Diagnosen. Für die Versorgung von Patient:innen mit diesen beiden Grunderkrankungen werden auch in anderen Untersuchungen Pandemieeffekte beschrieben [10, 23, 24]. Für den ambulanten Sektor berichten die kassenärztlichen Vereinigungen eine Verringerung der Konsultationen u. a. in kardiologischen und onkologischen Facharztpraxen um bis zu 40 % im Vergleich zum Vorjahr [2]. Für Personen mit Lungenerkrankungen und onkologische Patient:innen besteht ein besonders hohes Risiko für einen schweren Verlauf einer COVID-19-Erkrankung. Daher können die Terminabsagen durch die Versorgungseinrichtungen auch im Sinne von gezielten Infektionsschutzmaßnahmen seitens der Versorger:innen sowie als Hinweis auf einen durch Infektion und Quarantäne situativ verstärkten Fachkräftemangel interpretiert werden. In einer belgischen Studie [12] berichteten Ärzt:innen, dass sie Patient:innen aus diesen Hochrisikogruppen seit Beginn der COVID-19-Pandemie seltener einbestellen und stattdessen andere Wege der Versorgung, wie bspw. telemedizinische Konsultationen, suchten. Die Sicherung der Versorgung von chronisch Kranken durch alternative Strategien wird auch bestätigt durch den globalen WHO-Survey [22] sowie durch mündliche Mitteilungen aus dem Bereich der kassenärztlichen Versorgung Hamburg und ist zu interpretieren als Hinweis darauf, dass ein ausgesetzter Termin nicht mit einer nicht erfolgten Versorgungsleistung gleichzusetzen ist.

### Gründe für das Aussetzten von Versorgungsleistungen

Insgesamt sowie auch für die Untergruppe der Personen mit Vorerkrankungen wurden Termine häufiger von den Patient:innen selbst als von den Versorger:innen abgesagt; diese Tendenz wird durch die vergleichbare RKI-Studie [14] bestätigt. Als ein wesentlicher Grund wurde in der vorliegenden Studie die „Angst vor einer SARS-CoV-2-Infektion“ angeführt, die – wie auch Hajek et al. [19] diskutieren – bei aufgrund einer Vorerkrankung vulnerablen Personen ausgeprägter ist.

### Fachspezifische Versorgungsleistungen

In Übereinstimmung mit den Ergebnissen der RKI-Studie [14] wurden in der vorliegenden Studie deutlich häufiger fachärztliche als hausärztliche Versorgungsleistungen ausgesetzt. Auswertungen ambulanter Versorgungsdaten des Zentralinstituts der Kassenärztlichen Versorgung zeigen für das Jahr 2020 ebenfalls einen stärkeren Rückgang fachärztlicher als hausärztlicher Patient:innenkontakte, allerdings wurde in den Folgezeiträumen insbesondere für die fachärztliche Versorgung ein „Nachholeffekt“ beobachtet [11].

### Stärken und Limitation

Wesentliche Stärken dieser Studie liegen in ihrem direkten, bevölkerungsbezogenen Erhebungsansatz und in der differenzierten Analyse der Inanspruchnahme, die eine Unterscheidung nach patient:innen- und versorger:innenseitigen Terminabsagen ermöglicht. Die umfangreiche bevölkerungsbezogene Datenbasis der HCHS erlaubte eine separate Betrachtung von Personen mit und ohne chronische Erkrankungen.

Als Limitationen sind zu diskutieren: Zwischen der Erhebung der Inanspruchnahme während der COVID-19-Pandemie und der anamnestischen Erfassung chronischer Erkrankungen zum Zeitpunkt der Baseline-Untersuchung liegen teilweise längere Zeiträume von bis zu 6 Jahren. Auch wenn bei einem Großteil der hier betrachteten Erkrankungen eine Chronizität angenommen werden kann, fehlen Informationen zum aktuellen Gesundheitszustand sowie zum klinischen und subjektiv wahrgenommenen Versorgungsbedarf.

Eine Fehlklassifizierung hinsichtlich des Erkrankungsstatus kann nicht ausgeschlossen werden. Darüber hinaus sind in den Erkrankungsgruppen teilweise sehr unterschiedliche Diagnosen zusammengefasst und die Fallzahl ist, da es sich um eine Bevölkerungsstichprobe und kein Patient:innenkollektiv handelt, niedrig. Auch wenn die häufigsten „Volkskrankheiten“ eingeschlossen wurden, lagen zu einigen Diagnosen, wie z. B. Autoimmunerkrankungen, keine ausreichenden Diagnosedaten vor, so dass diese hier nicht berücksichtigt werden konnten. Insgesamt ist davon auszugehen, dass sich in der Gruppe der als „nicht chronisch erkrankt“ Klassifizierten auch Personen mit Vorerkrankungen befinden. In diesem Fall würde ein Unterschied zwischen den beiden Gruppen unterschätzt. Weiterhin handelt es sich um selbstberichtete Daten; auch wenn es keine entsprechenden Hinweise gab, ist ein Reportingbias nicht auszuschließen.

Bei der Interpretation der Daten ist zu berücksichtigen, dass ermittelt wurde, wie hoch der Anteil von Personen mit ausgesetzten Versorgungsleistungen ist, nicht jedoch die Anzahl und Art der ausgefallenen oder verschobenen Leistungen, so dass keine Aussagen dazu getroffen werden können, ob für Personen mit chronischen Erkrankungen eine ausreichende Versorgung gewährleistet war oder Versorgungslücken bestanden.

## Fazit

Erwachsene mit chronischen Grunderkrankungen sind vom Aussetzen gesundheitlicher Versorgungsleistungen in der COVID-19-Pandemie betroffen. Auch wenn mit ca. 80 % ein Großteil keine Einschränkungen berichtete, hat jede:r Fünfte mindestens einmal auf einen Arzt- oder Behandlungstermin verzichtet.

In Hinblick auf die Angst vor einer SARS-CoV-2-Infektion als ein Hauptgrund der Nichtinanspruchnahme stehen auch Gesundheitspolitik und Medien vor der Herausforderung, mit Ängsten vor einer Infektion so umzugehen, dass notwendige Versorgungsleistungen dadurch nicht vermieden werden. Ein engmaschiges Monitoring des Inanspruchnahmeverhaltens und Versorgungsgeschehens auch aus Patient:innenperspektive sowie longitudinale Analysen zu potenziellen gesundheitlichen Folgen bei Menschen mit chronischen Erkrankungen sind anzustreben.

## Supplementary Information





## References

[1] Gassen A (2020). Pandemie-Management in der ambulanten Versorgung: Analyse des bisherigen Verlaufs – Strategien und Maßnahmen für die Zukunft.

[2] Scheidt-Nave C, Barnes B, Beyer A-K, Busch MA, Hapke U, Heidemann C, Imhoff M (2020). Versorgung von chronischen Kranken in Deutschland – Herausforderungen in Zeiten der COVID-19-Pandemie. J Health Monit.

[3] Günster C, Drogan D, Hentschker C, Klauber J, Malzahn J, Schillinger G (2020). WIdO-Report: Entwicklung der Krankenhausfallzahlen während des Coronavirus-Lockdowns: Nach ICD-10-Diagnosekapiteln und ausgewählten Behandlungsanlässen: Wissenschaftliches Institut der AOK (WIdO).

[4] Metzler B, Siostrzonek P, Binder RK, Bauer A, Reinstadler SJ (2020). Decline of acute coronary syndrome admissions in Austria since the outbreak of COVID-19: the pandemic response causes cardiac collateral damage. Eur Heart J.

[5] Bitzer EM, Ansmann L, Hörold M, Lyssenko L, Apfelbacher C (2021). “I better stay at home…”-health system decisions to support the use of routine healthcare during the COVID-19 pandemic. Bundesgesundheitsblatt Gesundheitsforschung Gesundheitsschutz.

[6] Lee M, You M (2021). Avoidance of healthcare utilization in South Korea during the Coronavirus disease 2019 (COVID-19) pandemic. Int J Environ Res Public Health.

[7] Lehnert T, König H-H (2012). Effects of multimorbidity on health care utilization and costs. Bundesgesundheitsblatt Gesundheitsforschung Gesundheitsschutz.

[8] Deutsche Gesellschaft für Allgemeinmedizin und Familienmedizin, Zentralinstitut für die kassenärztliche Versorgung in der Bundesrepublik Deutschland (2020) Anforderungen an die Anforderungen an die Organisation der ambulanten hausärztlichen Versorgung während der COVID-19-Pandemie. https://www.degam.de/files/Inhalte/Degam-Inhalte/Ueber_uns/Positionspapiere/Zi_DEGAM_Versorgungsplanung_2020-05-06_final.pdf. Zugegriffen: 5. Sept. 2022

[9] Hacker KA, Briss PA, Richardson L, Wright J, Petersen R (2021). COVID-19 and chronic disease: the impact now and in the future. Prev Chronic Dis.

[10] Klauber J, Wasem J, Beivers A, Mostert C (2022). Krankenhaus-Report 2022: Patientenversorgung während der Pandemie.

[11] Mangiapane S, Zhu L, Kretschmann J, Czihal T, Stillfried D (2021) Veränderung der vertragsärztlichen Veränderung der vertragsärztlichen Leistungsinanspruchnahme während der COVID-Krise: Tabellarischer Trendreport für das Jahr 2020. https://www.zi.de/fileadmin/images/content/Publikationen/Trendreport_4_Leistungsinanspruchnahme_COVID_2021-04-19.pdf. Zugegriffen: 11. Juli 2022

[12] Danhieux K, Buffel V, Pairon A, Benkheil A, Remmen R, Wouters E (2020). The impact of COVID-19 on chronic care according to providers: a qualitative study among primary care practices in Belgium. BMC Fam Pract.

[13] Jagodzinski A, Johansen C, Koch-Gromus U, Aarabi G, Adam G, Anders S (2020). Rationale and design of the Hamburg city health study. Eur J Epidemiol.

[14] Heidemann C, Reitzle L, Schmidt C, Fuchs J, Prütz F, Scheidt-Nave C (2022). Nichtinanspruchnahme gesundheitlicher Versorgungsleistungen während der COVID-19-Pandemie: Ergebnisse der CoMoLo-Studie. J Health Monit.

[15] (2021) Epidemiologischer Steckbrief zu SARS-CoV‑2 und COVID-19. https://www.rki.de/DE/Content/InfAZ/N/Neuartiges_Coronavirus/Steckbrief.html?nn=13490888#doc13776792bodyText21. Zugegriffen: 25. 2022

[16] Cummings MJ, Baldwin MR, Abrams D, Jacobson SD, Meyer BJ, Balough EM (2020). Epidemiology, clinical course, and outcomes of critically ill adults with COVID-19 in New York City: a prospective cohort study. Lancet.

[17] UNESCO Institute for Statistics (2012). International Standard Classification of Education ISCED 2011.

[18] Splinter MJ, Velek P, Ikram MK, Kieboom BCT, Peeters RP, Bindels PJE (2021). Prevalence and determinants of healthcare avoidance during the COVID-19 pandemic: A population-based cross-sectional study. PLoS Med.

[19] Hajek A, de Bock F, Kretzler B, König H-H (2021). Factors associated with postponed health checkups during the COVID-19 pandemic in Germany. Public Health.

[20] Heidemann C, Paprott R, Huebl L, Scheidt-Nave C, Reitzle L (2020). Selbst eingeschätzte medizinische Versorgung im Verlauf der SARS-CoV-2-Pandemie in Deutschland: Ergebnisse der COSMO-Studie.

[21] Du Y, Baumert J, Damerow S, Rommel A, Scheidt-Nave C, Heidemann C (2021). Inanspruchnahme ambulanter ärztlicher Leistungen während der COVID-19-Pandemie bei Personen mit und ohne Diabetes in Deutschland. J Health Monit.

[22] World Health Organization (2020). The impact of the COVID-19 pandemic on noncommunicable disease resources and services: results of a rapid assessment.

[23] Dinmohamed AG, Visser O, Verhoeven RHA, Louwman MWJ, van Nederveen FH, Willems SM (2020). Fewer cancer diagnoses during the COVID-19 epidemic in the Netherlands. Lancet Oncol.

[24] Chudasama YV, Gillies CL, Zaccardi F, Coles B, Davies MJ, Seidu S (2020). Impact of COVID-19 on routine care for chronic diseases: A global survey of views from healthcare professionals. Diabetes Metab Syndr.

