# Supplementary material for: Non-utilisation of medical services during the COVID-19 pandemic among persons with chronic diseases
Source: Bundesgesundheitsblatt Gesundheitsforschung Gesundheitsschutz. 2023 Feb 1;66(3):275–82. [Article in German] doi: 10.1007/s00103-023-03665-9 (PMC9891187; doi:10.1007/s00103-023-03665-9)
Supplement: Supplementary file 1 [file 103_2023_3665_MOESM1_ESM.pdf]

Onlinematerial 1: Vorbestehende Erkrankungen: Prävalenz und Definition (n=857 Personen mit Vorerkrankungen)

**Vorerkrankung (Prävalenz in der Studienpopulation); n=857 Personen mit Vorerkrankungen**	**Zugehörige Diagnosen**	**Definition in der HCHS**
**Lungenerkrankungen (16,2 %)**	Chronisch-obstruktive Lungenerkrankungen	Anamnestische Selbstangabe
	Asthma bronchiale	Anamnestische Selbstangabe
**Herzerkrankungen (10,0 %)**	Myokardinfarkt	Anamnestische Selbstangabe
	Herzinsuffizienz	Anamnestische Selbstangabe
	Koronare Herzerkrankung	Anamnestische Selbstangabe
	Angina pectoris	Anamnestische Selbstangabe
	Vorhofflimmern	Anamnestische Selbstangabe oder Vorhofflimmern im 12-Kanal-EKG während der Basis-Untersuchung
**Chronische Nierenerkrankungen (10,4 %)**	Chronische Nierenerkrankungen allgemein	Anamnestische Selbstangabe
**Krebserkrankungen (9,8%)**	Krebserkrankungen jemals	Anamnestische Selbstangabe
**Diabetes mellitus (7,1 %)**	Diabetes mellitus	Anamnestische Selbstangabe oder Nüchternglukose >126 mg/dl oder Glukose >200 mg/dl oder Medikation mit Insulin
**Demenz (3,3 %)**	Demenz	Anamnestische Selbstangabe oder Mini-Mental-Status-Test <25 Punkte
**Schlaganfall (1,5 %)**	Schlaganfall	Anamnestische Selbstangabe

Erhebung der vorbestehenden Erkrankungen

Die Informationen (anamnestische Angaben/Untersuchungen) zu den hier berücksichtigten Vorerkrankungen wurden für alle Teilnehmer:innen der Hamburg City Health Study (HCHS) im Rahmen des Standard HCHS-Programms über anamnestische Interviews und/oder Untersuchungen generiert.

*Anamnestische Selbstangaben wurden dabei erhoben über die Frage: „Wurde bei Ihnen jemals ärztlich eine/ein *DIAGNOSE* festgestellt?“*
